# Long non-coding RNA expression profile in human gastric cancer and its clinical significances

**DOI:** 10.1186/1479-5876-11-225

**Published:** 2013-09-24

**Authors:** Haojun Song, Weiliang Sun, Guoliang Ye, Xiaoyun Ding, Zhong Liu, Sijie Zhang, Tian Xia, Bingxiu Xiao, Yang Xi, Junming Guo

**Affiliations:** 1 Department of Biochemistry and Molecular Biology, and Zhejiang Provincial Key Laboratory of Pathophysiology, Ningbo University School of Medicine, Ningbo 315211, China; 2 Ningbo Yinzhou People’s Hospital and the Affiliated Hospital, Ningbo University School of Medicine, Ningbo 315040, China; 3 The Affiliated Hospital, Ningbo University School of Medicine, Ningbo 315010, China; 4 Ningbo No. 1 Hospital and the Affiliated Hospital, Ningbo University School of Medicine, Ningbo 315010, China

**Keywords:** Gastric cancer, Long non-coding RNA, Expression profile, H19, uc001lsz

## Abstract

**Background:**

Long non-coding RNAs (lncRNAs) are prevalently transcribed in the genome yet their potential roles in human cancers are not well understood. The aim of the present study was to determine the lncRNA expression profile in gastric cancer and its potential clinical value.

**Methods:**

The global lncRNA expression profile in gastric cancer was measured by lncRNA microarray. Levels of two representative lncRNAs, H19 and uc001lsz, were confirmed by real-time reverse transcriptase-polymerase chain reaction. The relationship between their levels and clinicopathological factors of patients with gastric cancer was explored. A receiver operating characteristic (ROC) curve was constructed for differentiating gastric cancer from benign gastric diseases.

**Results:**

Total of 135 lncRNAs, which differential expression levels between tumor and non-tumorous tissues were more than twofold, were found (GEO No. GSE47850). The most down-regulated lncRNAs in gastric cancer tissues were FER1L4, uc001lsz, BG491697, AF131784, uc009ycs, BG981369, AF147447, HMlincRNA1600, and AK054588; while the most up-regulated ones were H19, HMlincRNA717, BM709340, BQ213083, AK054978, and DB077273. H19 was found highly expressed in stomach and liver cancer cell lines, while lowly expressed in lung cancer and prostate cancer cell lines. Uc001lsz was lowly expressed in gastric, lung and liver cancer cell lines, while highly expressed in prostate cancer. The areas under ROC curves were up to 0.613, 0.751, and 0.761 for H19, uc001lsz, and the combination, respectively.

**Conclusions:**

The lncRNA expression profile in gastric cancer suggests the potential roles of lncRNAs in gastric cancer occurrence and development. The overexpression of H19 in gastric cancer suggests that H19 may be participated in gastric cancer. The reduced expression of uc001lsz in gastric cancer cell lines and tissues, its associations with TNM stage, and its dysregulation in early cancer and precancerous lesions suggest that uc001lsz may be a potential marker for the diagnosis of early gastric cancer.

## Background

The well-studied components in the human genome are those of protein-coding genes. However, the coding exons of these genes account for only 1.5% of the genome [1]. In recent years, it has become increasingly apparent that the non-protein-coding portion of the genome is of crucial functional importance for disease occurrence [2]. The non-coding RNAs (ncRNAs) characterize as three types, long ncRNAs, mid-size ncRNAs and short ncRNAs [1]. Although most studies on ncRNAs are focused on short ncRNAs, such as microRNAs (miRNAs), long non-coding RNAs (lncRNAs) are rapidly gaining prominence recently.

LncRNAs are greater than 200 nucleotides in length [3]. They have emerged recently as major players in governing fundamental biological processes. Aberrant expression of lncRNAs has been associated with cancers [3]. For example, Differential display code 3 (DD3^PCA3^), a prostate-specific lncRNA, appears to be a marker for early diagnosis of prostate cancer [4]. More important, DD3^PCA3^ can be detected in urine from patients with prostate cancer [5]. Though Metastasis associated lung adenocarcinoma transcript 1 (MALAT-1) is first found abnormal expressed in metastasizing non-small-cell lung carcinomas [6], it is up-regulated in hepatocarcinoma, breast cancer, pancreatic cancer, colorectal cancer, and prostate cancer [7]. MALAT-1 is not only a potential diagnostic marker, but also a potential prognostic marker [8]. HOX transcript antisense RNA (HOTAIR) is associated with breast cancer and colorectal cancer [9,10]. H19, another famous lncRNA, is frequently involved in pediatric and adult tumors [11].

Gastric cancer is still one of the most frequent causes of mortality in the world [12]. However, traditional strategies based on radical surgery for the treatment of gastric cancer are not yet satisfactory. Therefore, reveal of the mechanisms of occurrence and development of gastric cancer is attracting increased attention in cancer research.

Since the global lncRNA expression profile in gastric cancer is not fully uncovered, in the present study, we explored the lncRNA expression profile in gastric cancer. Then the relationship between the aberrantly expressed-lncRNAs and clinicopathological factors of patients with gastric cancer was explored. Our data provides candidate diagnostic biomarkers of gastric cancer.

## Methods

### Patients and specimens

Gastric cancer patients’ tissues, including gastric cancer tissues, precancerous lesion and corresponding adjacent non-tumorous tissues were immediately preserved in RNA fixer (Bioteke, Beijing, China) after removal from the body and stored at –80°C until use. Tissue samples were obtained from surgical or biopsy specimens from February 2011 to June 2012 at three cancer centers, Yinzhou People’s Hospital, Ningbo No. 1 Hospital and The Affiliated Hospital of Ningbo University School of Medicine, China. Informed consent was taken from all subjects. The Human Research Ethics Committee of Ningbo University approved all aspects of this study. Tumors were staged according to the tumor-node-metastasis (TNM) staging system of the International Union Against Cancer (5^th^ ed). Histological grade was assessed following the National Comprehensive Cancer Network (NCCN) clinical practice guideline of oncology (V.1.2011). The non-tumorous tissues were 5 cm from the edge of the tumor and there were no obvious tumor cells, as evaluated by a pathologist. There was no radiotherapy, chemotherapy, targeted therapy or Dendritic cell/Cytokine-induced killer (DC/CIK) therapy prior to the upper gastrointestinal endoscopy examination or operation.

### Total RNA preparation

Total RNA was isolated using Trizol reagent (Invitrogen, Karlsruhe, Germany) following the manufacturer’s instructions [13].

### LncRNA microarray assay

Three paired biopsy specimens were obtained from patients (55 y and male, 76 y and male, and 88 y and female) with poorly or poorly-and-moderately differentiated gastric cancer. Human lncRNA microarray was manufactured in NimbleGen Hybridization System (Arraystar, Rockville, MD). More than 23 000 lncRNAs were collected from the authoritative data sources including National Center for Biotechnology Information (NCBI) RefSeq, University of California, Santa Cruz (UCSC), lncRNAs from literatures and Ultra Conserved Regions (UCRs) [14]. Data was extracted and normalized using NimbleScan v2.5 software (Roche NimbleGen, Madison, WI). Further Data analysis was performed using Agilent GeneSpring GX 11.5 software (Agilent Technologies, Santa Clara, CA).

### Cell culture

Human gastric epithelial cell line (GES-1), gastric cancer cell lines (AGS, MGC-803, and SGC-7901), liver normal cell line (HL-7702), hepatic carcinoma cell lines (HepG2 and SMMC-7721), fetal lung fibroblast cell line (HELF), lung carcinoma cell line (A549), prostate epithelial cell line (RWPE-1), and prostate carcinoma cell lines (Du-145 and PC-3) were obtained from the Shanghai Institute of Biochemistry and Cell Biology, Chinese Academy of Sciences (Shanghai, China). Cells were cultured in culture flasks at 37°C in a humidified atmosphere of 5% CO_2_[15].

### qRT-PCR detection of H19 and uc001lsz

Real-time quantitative reverse transcription-polymerase chain reaction (qRT-PCR) is the gold standard for data verification. To verify the results of lncRNA microarray, cDNA was generated using the GoScript Reverse Transcription (RT) System (Promega, Madison, WI). Quantitative polymerase chain reaction (qPCR) was achieved by using the GoTaq qPCR Master Mix (Promega, Madison, WI) on an Mx3005P real-time PCR System (Stratagene, La Jolla, CA). The sequences of the PCR primers for H19, uc001lsz, and β-actin were as follows: 5′-ACCAGCCACCACATCATC-3′ (sense) and 5′-TCAGAAACAAAGAGACAGAAGG-3′ (antisense) for H19; 5′-GACGGCACCTACTACACCTT-3′ (sense) and 5′-GCTGACCACCTTGTTGTTGAA-3′ (antisense) for uc001lsz; 5′-AAGCCACCCCACTTCTCTCTAA-3′ (sense) and 5′-AATGCTATCACCTCCCCTGTGT-3′ (antisense) for β-actin. The data were analyzed by the ∆*C*_t_ method [16,17]. All results were expressed as the Means ± SD of three independent experiments.

### Cloning and sequencing of qRT-PCR products

The qRT-PCR products of lncRNA were first purified using a UNIQ-10 PCR Product Purification Kit and then cloned into the pUCm-T vector (Sangon Biotech, Shanghai, China) following the manufacturer’s instructions. Then, DNA sequencing was performed by Sangon Biotech Co., Ltd.

### Serological tumor marker analysis

Serum carcinoembryonic antigen (CEA) and carbohydrate antigen 19-9 (CA19-9) were measured using an Elecsys 2010 machine (Roche Diagnostics, Basel, Switzerland). The cutoff values were 5 ng/mL and 35 U/mL for CEA and CA19-9, respectively.

### Statistical analysis

All statistical data were analyzed by Statistical Program for Social Sciences (SPSS) 18.0 software (SPSS, Chicago, IL) and GraphPad Prism 6.0 (GraphPad Software, La Jolla, CA). One way analysis of variance test, two-tailed Student’s *t*-test and rank-sum test were used as appropriate. A receiver operating characteristic (ROC) curve was established to evaluate the diagnostic value. *P* < 0.05 was considered statistically significant.

## Results

### LncRNA expression profiles in gastric cancer tissues relative to adjacent non-tumorous tissues

**Figure 1:**
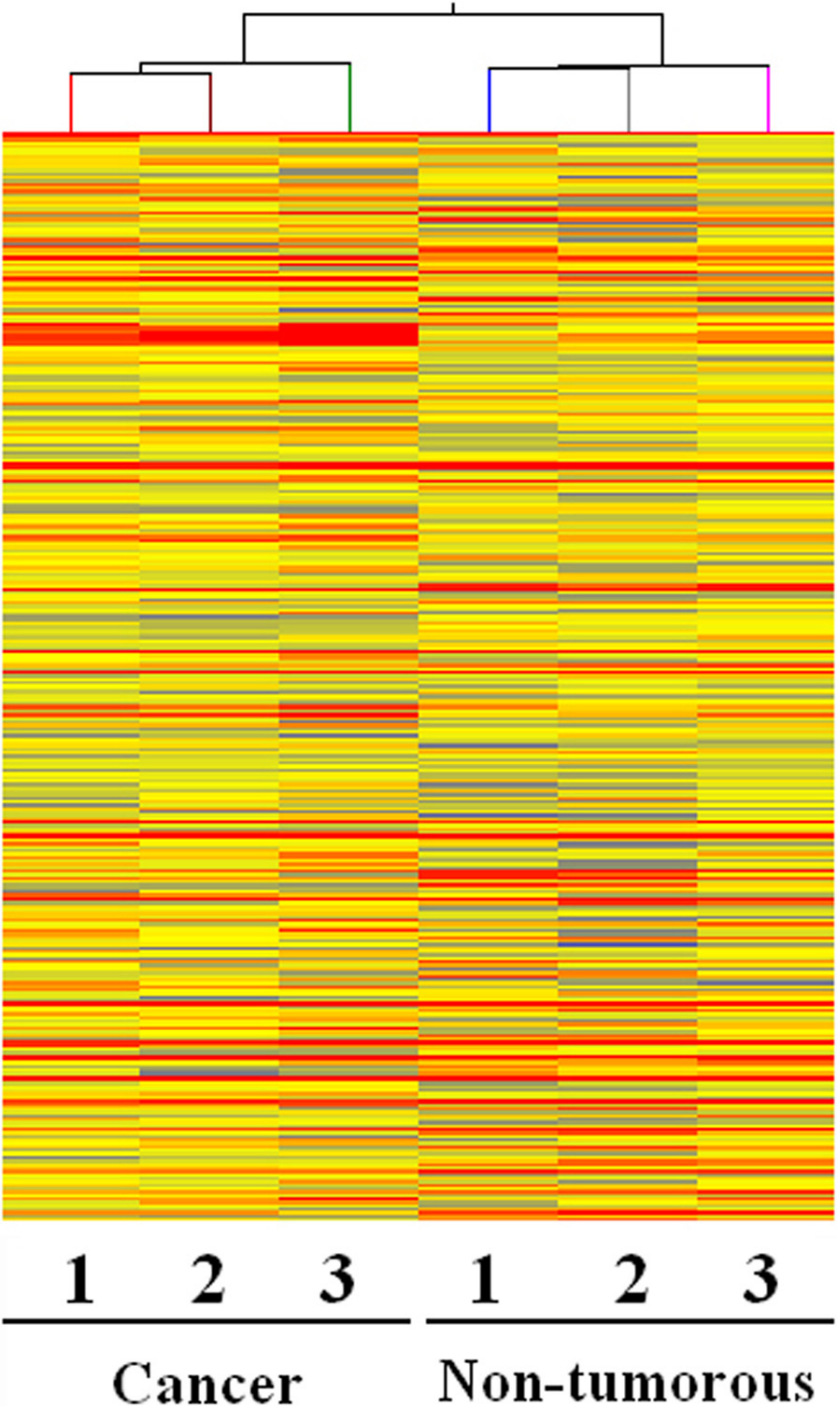
**Alterations in lncRNA expression profiles between gastric carcinoma tissues and non-tumorous tissues.** The result from Hierarchical Clustering shows distinguishable lncRNA expression profiling among samples. “Red” indicates high relative expression; and “blue” indicates low relative expression.

The lncRNA expression patterns between gastric cancer tissues and adjacent non-tumorous tissues were found to be significantly different (Figure 1). Total of 135 lncRNAs, which expression change was more than twofold, were found (GEO accession numbers is 47850; http://www.ncbi.nlm.nih.gov/geo/query/acc.cgi?acc=GSE47850). Among them, 71 and 64 were up and down expressed in tumor tissues, respectively. The most down-regulated lncRNAs in gastric cancer tissues were FER1L4, uc001lsz, BG491697, AF131784, uc009ycs, BG981369, AF147447, HMlincRNA1600, and AK054588. The most up-regulated lncRNAs in gastric cancer tissues were H19, HMlincRNA717, AI769947, BQ213083, AK054978, and DB077273 (Table 1).

**Table 1:** More than fourfold differentially expressed lncRNAs in gastric cancer tissues comparing with paired non-tumorous tissues

**Name**	**Chromosome**	**Regulation**	**Fold change**	**Source**^**a**^	***P *value**
H19	11	up	8.91	lncRNAdb	0.020
HMlincRNA717	18	up	5.96	lincRNA	0.013
AI769947	7	up	5.51	lincRNA	0.026
BQ213083	2	up	5.45	lincRNA	0.040
AK054978	X	up	4.59	lincRNA	0.006
DB077273	2	up	4.11	lincRNA	0.008
FER1L4	20	down	9.17	refNR	0.047
uc0 01lsz	11	down	8.36	UCSC_knowngene	0.020
BG491697	20	down	6.50	lincRNA	0.035
AF131784	18	down	6.25	mRNA	0.019
uc009ycs	11	down	5.82	UCSC_knowngene	0.009
BG981369	1	down	5.20	lincRNA	0.048
AF147447	16	down	4.76	mRNA	0.038
HMlincRNA1600	X	down	4.12	lincRNA	0.013
AK054588	1	down	4.04	lincRNA	0.045

^a^lncRNAdb: http://lncrnadb.com/Default.aspx; lincRNA: http://genome.ucsc.edu/cgi-bin/hgGateway; refNR: http://genome.ucsc.edu/cgi-bin/hgTables; http://www.ncbi.nlm.nih.gov/RefSeq/; UCSC_knowngene: http://genome.ucsc.edu/cgi-bin/hgTables; mRNA: http://genome.ucsc.edu/cgi-bin/hgTables.

### Expression of H19 was up-regulated in gastric carcinoma tissues

**Figure 2:**
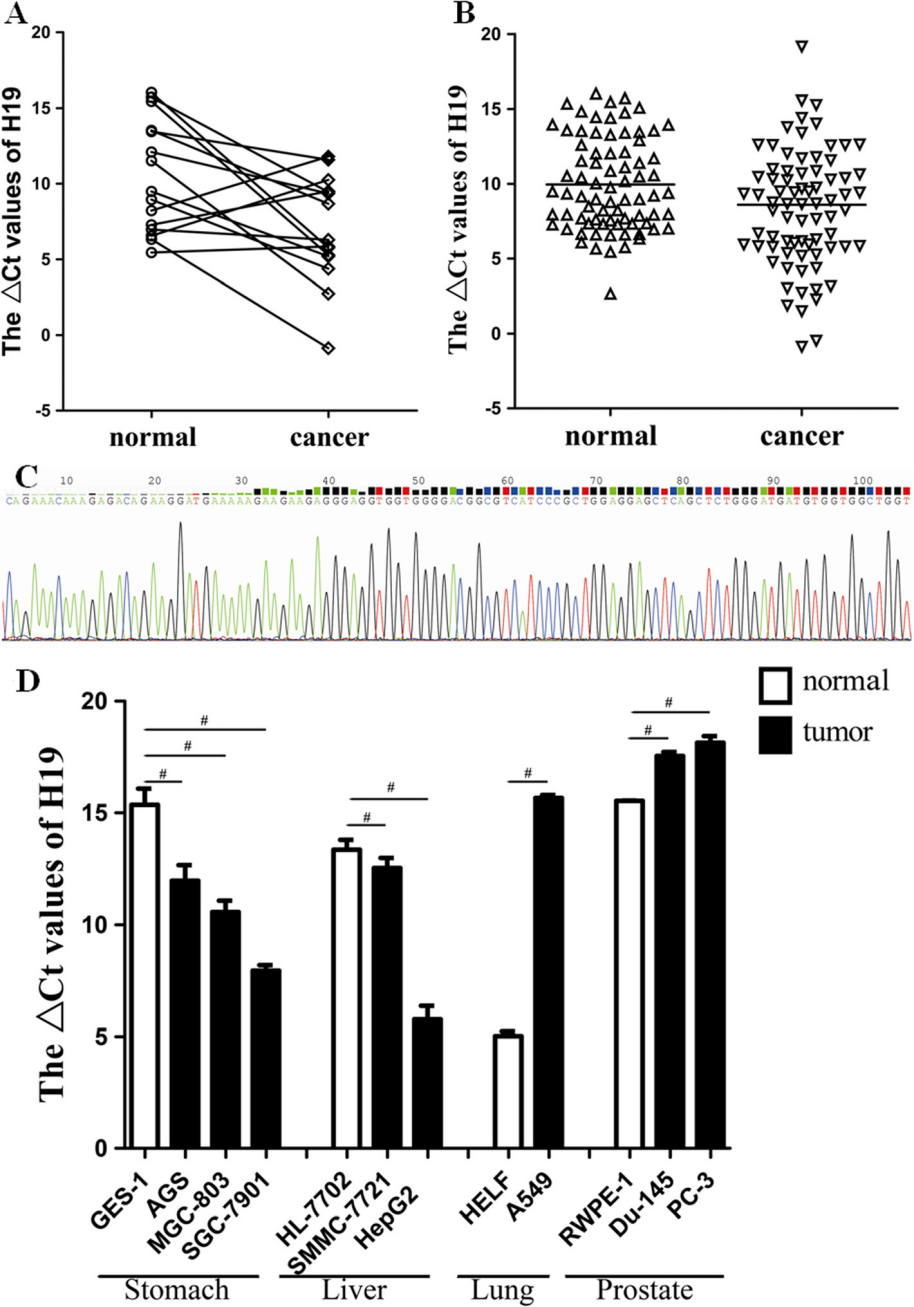
**H19 was up-regulated in gastric cancer tissues, gastric cancer cell lines and other common cancer cell lines.** Real-time qRT-PCR was used to determine the expression level of H19. The ∆*C*_t_ value was determined by subtracting the β-actin *C*_t_ value from the target lncRNA *C*_t_ value. Smaller ∆*C*_t_ value indicates higher expression. Level of H19 in gastric cancer biopsy tissues (*n* = 15, *P* = 0.014; **A**) and gastric cancer tissues (*n* = 77, *P* = 0.029; **B**). Sequencing result of qRT-PCR product of H19 **(C)**. The level of H19 in gastric cancer cell lines (AGS, MGC-803 and SGC-7901) and liver cancer cell lines (SMMC-7721 and HepG2) was higher than that in human gastric epithelial cell line GES-1 and human liver normal cell line HL-7702, respectively; however, its level in lung cancer cell line A549 and prostate cancer cell lines (Du-145 and PC-3) was lower than that in human fetal lung fibroblast cell line HELF cells and human prostate epithelial cell line RWPE-1 **(D)**. All results were expressed as the Means ± SD of three independent experiments. ^#^*P* < 0.001.

Since H19 was found the most up-regulated lncRNA in gastric cancer tissue, up to 8.91-fold change in microarray detection (Table 1), to validate this result, we detected the expression level of H19 in two types of cancer tissues, biopsy tissues and surgical specimens, by qRT-PCR. First, we explored the expression level of H19 in 15 pairs of gastric carcinoma’ biopsy tissues. We found that its level in cancer tissues was significantly higher than that in non-tumorous tissues (Figure 2A). Then we detected H19 level in large number of surgical specimens. As shown in Figure 2B, it was up-regulated in 74.0% (57/77) of gastric cancer tissues (*P* = 0.029). By sequencing the qRT-PCR product, we found that the sequence of H19 (Figure 2C) was consistent with that from the database (http://www.ncbi.nlm.nih.gov/gene/283120).

### Expression of H19 in common cancer cell lines

To obtain more information about H19 expression in cancers, we investigated its level in some common cancer cell lines. As shown in Figure 2D, comparing with respective normal cell line, H19 was found highly expressed in stomach cancer cell lines (AGS, MGC-803 and SGC-7901) and hepatocellular carcinoma cell lines (SMMC-7721 and HepG2), while lowly expressed in lung cancer cell line (A549) and prostate cancer cell lines (Du-145 and PC-3).

### Expression of uc001lsz was down-regulated in gastric carcinoma tissues

**Figure 3:**
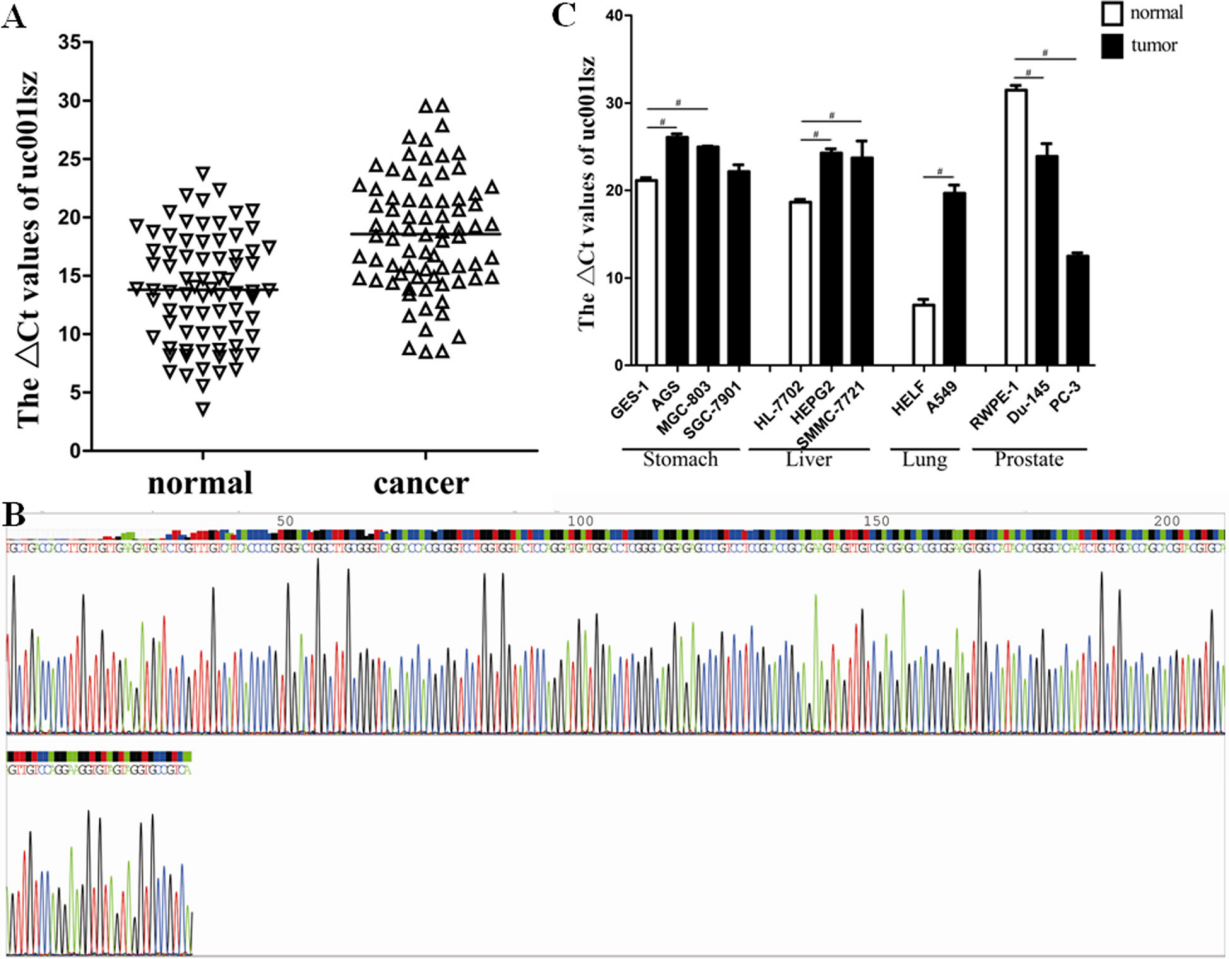
**Uc001lsz was down-regulated in gastric cancer tissues, gastric cancer cell lines and other common cancer cell lines.** Real-time qRT-PCR was used to determine the expression level of uc001lsz. Three independent experiments were performed. Level of uc001lsz in gastric cancer tissues was significantly lower than that in corresponding non-tumorous tissues (*n* = 77, *P* < 0.001; **A**). Sequencing result of qRT-PCR product of uc001lsz **(B)**. The level of uc001lsz in gastric cancer cell lines (AGS, MGC-803 and SGC-7901), liver cancer cell lines (SMMC-7721 and HepG2) and lung cancer cell line A549 was lower than that in human gastric epithelial cell line GES-1, human liver normal cell line HL-7702 and human fetal lung fibroblast cell line HELF, respectively; however, its level in prostate cancer cell lines (Du-145 and PC-3) was higher than that in human prostate epithelial cell line RWPE-1 **(C)**.

Uc001lsz is a new-found lncRNA, which is transcribed from the forward strand in chromosome 11p15.5. From the microarray results (Table 1), we can see that uc001lsz was the second most down-regulated lncRNA in gastric cancer tissue. Another reason that encouraged us to further study uc001lsz was that it is *trans* associated with MUC2, which is secreted and forms an insoluble mucous barrier in the gut lumen. To further validate the expression of uc001lsz in gastric cancer, we expanded the sample number. The data indicate that it was significantly down-regulated in 84.4% (65/77) of gastric cancer tissues (Figure 3A, *P* < 0.001). By sequencing the qRT-PCR product, we found that the sequence of uc001lsz (Figure 3B) was consistent with that from the database (http://genome.ucsc.edu/cgi-bin/hgTables).

### Expression of uc001lsz in common cancer cell lines

To obtain the expression information about uc001lsz in common cancers, we detected its level in several common cancer cell lines. We found that comparing with respective normal cell line, uc001lsz was lowly expressed in gastric cancer (AGS, MGC-803 and SGC-7901), lung cancer (A549) and liver cancer (SMMC-7721 and HepG2) cell lines, while only highly expressed in prostate cancer (Du-145 and PC-3) cell lines (Figure 3C).

### Potential diagnostic values of H19 and uc001lsz

**Figure 4:**
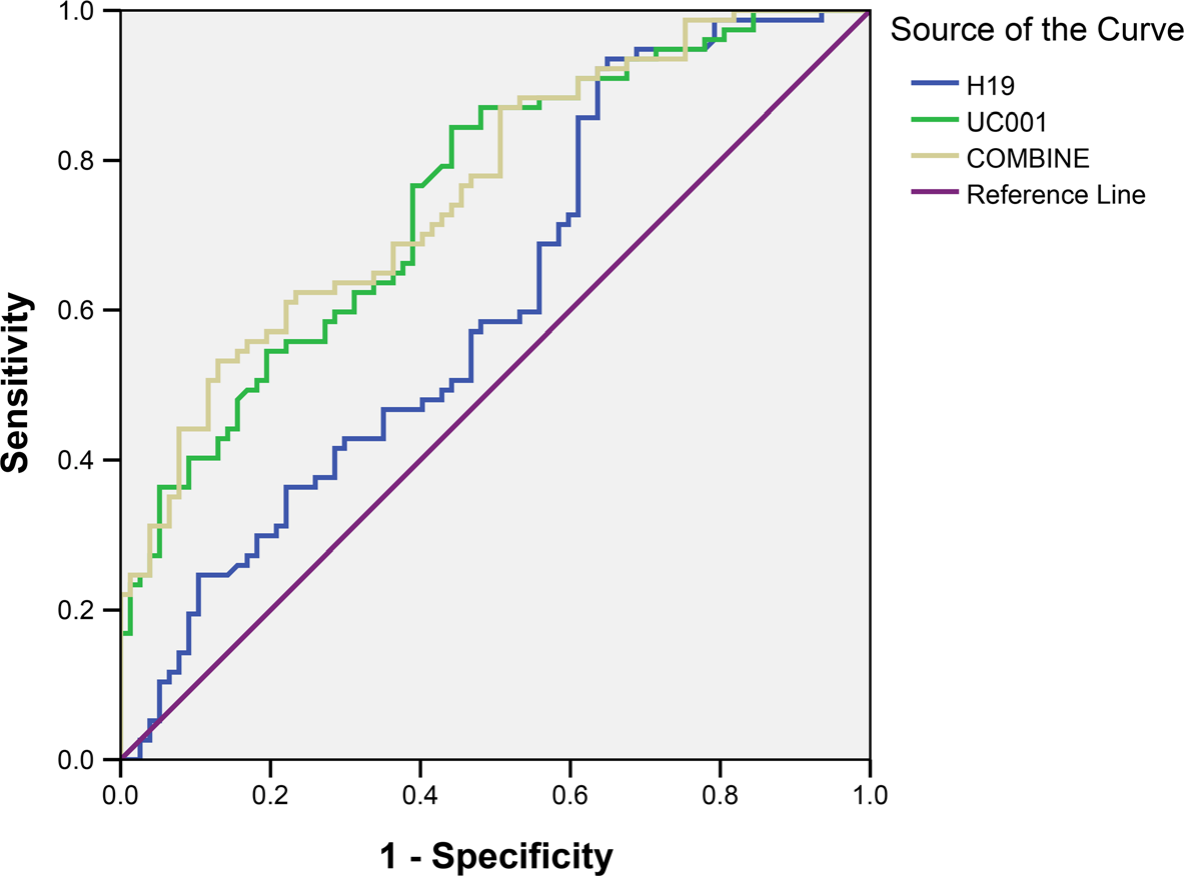
The ROC curve.

We next performed an analysis to identify whether H19 or uc001lsz expression was associated with the clinicopathological features of gastric cancer. As shown in Table 2, the level of uc001lsz was associated with TNM stages (*P* = 0.032). The positive detection rates of H19 and uc001lsz are 74.0% and 84.4%, respectively. Both of them are higher than those of common gastric cancer biomarker CEA (64.0%) and CA19-9 (53.3%) (Table 2). The areas under ROC curves were up to 0.613, 0.751, and 0.761 for H19, uc001lsz, and the combination, respectively (Figure 4). The combinative use of H19 and uc001lsz slightly increased the diagnostic value.

**Table 2:** **The relationship of H19 and uc001lsz expression levels (Δ*C***_**t**_**) in cancer tissues with clinicopathological factors of patients with gastric cancer**^**a**^

**Characteristics**	**No. of patients (%)**	**H19**		**uc001lsz**	
		**Mean ± SD**	***P *value**	**Mean ± SD**	***P *value**
Age (y)					
≥ 60	59 (76.6)	8.74 ± 4.67	0.588	18.85 ± 4.94	0.240
< 60	18 (23.4)	8.08 ± 3.99		17.28 ± 4.85	
Gender					
Male	57 (74.0)	8.66 ± 4.81	0.823	18.19 ± 5.01	0.355
Female	20 (26.0)	8.40 ± 3.44		19.37 ± 4.58	
Diameter (cm)^b^					
≥ 5	39 (52.0)	7.98 ± 4.44	0.282	18.49 ± 5.03	0.682
< 5	36 (48.0)	9.10 ± 4.53		18.01 ± 5.11	
CEA^b^					
Positive	48 (64.0)	8.45 ± 4.21	0.882	18.28 ± 4.54	0.593
Negative	27 (36.0)	8.61 ± 5.01		18.92 ± 5.71	
CA19-9^b^					
Positive	40 (53.3)	8.68 ± 3.87	0.720	18.65 ± 4.64	0.792
Negative	35 (46.7)	8.31 ± 5.14		18.35 ± 5.37	
Differentiation^c^					
Well	3 (4.9)	8.28 ± 1.92	0.635	15.34 ± 5.40	0.371
Moderate	33 (54.1)	9.05 ± 4.40		19.37 ± 4.85	
Poor	25 (41.0)	7.84 ± 5.47		19.54 ± 4.91	
Lymphatic metastasis^c^					
N0	20 (32.8)	8.95 ± 3.17	0.926	18.55 ± 6.01	0.472
N1&N2&N3	41 (67.2)	9.07 ± 5.25		19.53 ± 4.39	
Distal metastasis^c^					
M0	58 (95.1)	8.87 ± 4.70	0.258	19.13 ± 5.02	0.617
M1	3 (4.9)	12.00 ± 1.30		20.62 ± 3.74	
Invasion^c^					
Tis&T1-T3	20 (32.8)	10.04 ± 3.86	0.237	17.73 ± 4.72	0.105
T4	41 (67.2)	8.53 ± 4.95		19.93 ± 4.95	
TNM stage^c^					
0&I&II	24 (39.3)	9.40 ± 3.86	0.620	17.81 ± 5.52	0.032
III&IV	37 (60.7)	8.79 ± 5.14		20.49 ± 3.98	

^a^All samples are consist of 16 endoscopic biopsy samples and 61 surgery samples from patients with gastric cancer.

^b^Not detected for 2 patients who were performed endoscopy examination.

^c^Only includes 61 surgery patients.

### Expression of uc001lsz was aberrant in early cancer and precancerous lesions

**Figure 5:**
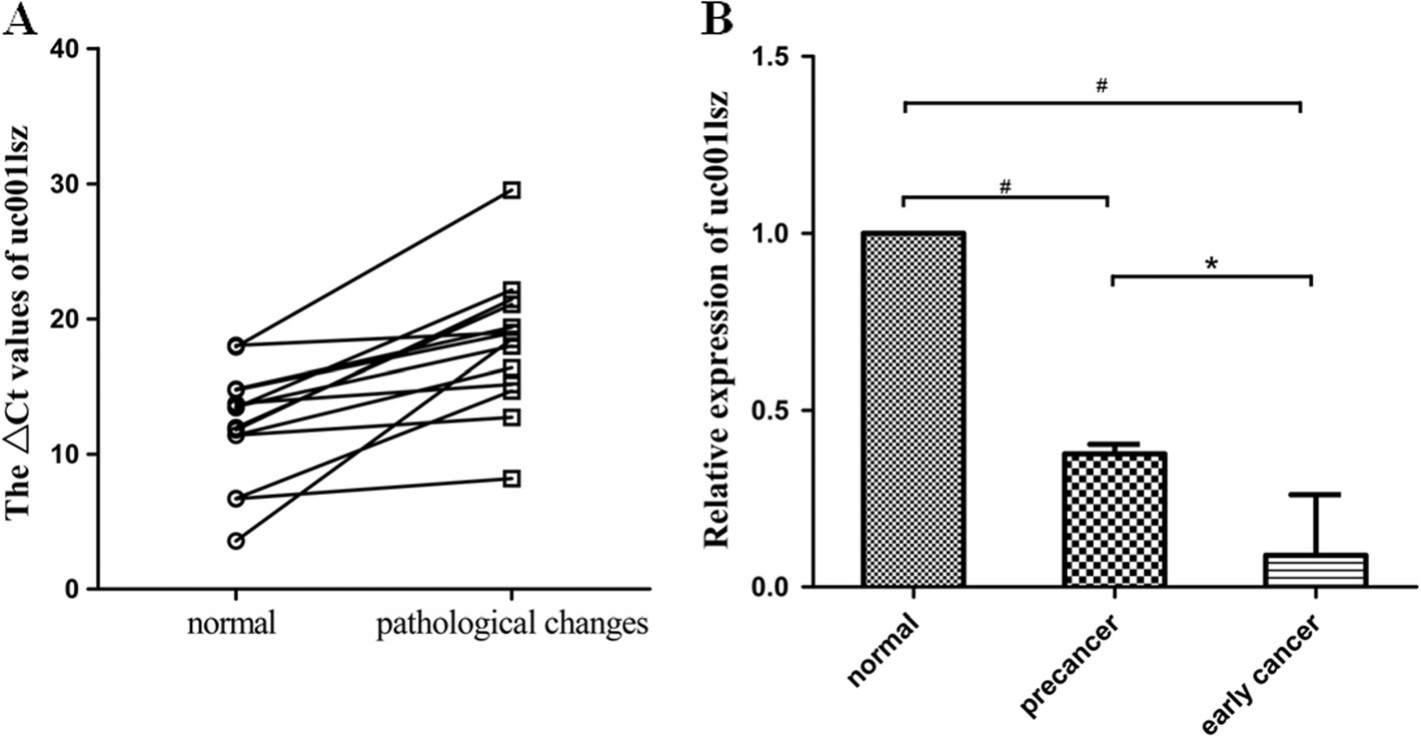
**Expression of uc001lsz in early cancer and precancerous lesions.** The level of uc001lsz in early pathological change tissue is lower than that in paired normal tissue (*n* = 14, *P* = 0.0016; **A**). The level of uc001lsz in normal tissue is significantly higher than that in precancerous lesions and early cancers **(B)**. ^#^*P* < 0.001; ^*^P < 0.05.

At last, to observe the possible early diagnostic values of lncRNA, we measured the level of uc001lsz in early cancer and precancerous lesions. We found that its level was remarkable lower in these lesions compared with those of corresponding adjacent non-tumorous tissues (Figure 5A). The level of uc001lsz in normal tissues was significantly higher than that in precancerous lesions and early cancer tissues. Besides, its level in precancerous lesions was conspicuous higher than that in early cancer tissues (Figure 5B).

## Discussion

Studies have shown that ~18% of the protein coding genes that produce lncRNAs are associated with cancer, whereas only 9% of all human protein coding genes are associated with cancer [18]. The relationship between lncRNAs and tumors has currently become one of the focuses of cancer studies.

There is mounting evidence that lncRNAs are relation to digestive system tumors [19]. Three lncRNAs, H19, HOTAIR and lncRNA highly upregulated in liver cancer (HULC), were found overexpression in human hepatocellular carcinomas (HCC) [20-22]. In addition, HULC expression is not only confined to HCC, but is also expressed in colorectal carcinomas that metastasize to the liver [23].

Researches about lncRNAs related to stomach are limited. Sun *et al.* found that the expression level of gastric cancer-associated transcript 1 (GACAT1), or AC096655.1-002, was significantly correlated with lymph node metastasis, distant metastasis, TNM stages, and differentiation [17]. Mei *et al.* reported that ubiquitin-like modifier (SUMO) 1 pseudogene 3, SUMO1P3, might be a potential biomarker in the diagnosis of gastric cancer [16]. Niinuma *et al.* found that overexpression of HOTAIR was markedly associated with high-risk gastrointestinal stromal tumors [24]. RNA, 7SK small nuclear (RN7SK) can indirectly regulate gastric tumorigenesis via positive transcription elongation factor-b (p-TEFb) [25]. H19 may play an important role in gastric cancer by loss of imprinting and other mechanisms [26,27]. Recent, Cao *et al*. used bioinformatics methods to screen lncRNA expression profiles associated with gastric cancer [28]. First, two publicly available human exon arrays for gastric cancer and data for the corresponding normal tissue were downloaded from the GEO. Then, the probes of the human exon arrays were re-annotated. Finally, the probes uniquely mapping to lncRNAs at the gene level were retained. Total of 88 lncRNAs that were differentially expressed in gastric cancer were identified [28].

Here, the approaches for the screening of gastric cancer–associated lncRNAs were different from those used by Cao *et al*. [28]. To identify the remarkably down-regulated or up-regulated lncRNAs in gastric cancer, we first collected gastric cancer and adjacent non-tumorous tissue samples from upper gastrointestinal endoscopy examination. From the lncRNA expression profiles obtained from lncRNA microarray analysis (Figure 1), we found that among the significantly different expressed lncRNAs (Table 1), only H19 has been found in other cancers [11,20,26,27]. As a result, it is crucial to further clarify the clinical signatures of lncRNAs in the diagnosis and treatment of gastric cancer.

The growing studies of functionally characterized lncRNAs reveals that these transcripts are important in different physiological processes, including embryonic stem cell differentiation [29], T-cell differentiation [30], keratinocyte differentiation [31], especially, the altered expression of lncRNAs could result in cancer [32].

In the present study, we focused on two lncRNAs, H19 and uc001lsz. The expression level of H19 in gastric cancer tissues was found to be evidently higher than that in non-tumor tissues (Figure 2A and B). Together with the increased expression of H19 in gastric cancer cell lines (Figure 2D), we suggest that H19 may play an important role in gastric cancer pathogenesis. Interesting, H19 expression is not increased in every types of tumor. As showed in Figure 2D, H19 expression is decreased in hepatocarcinoma and prostate cancer. These results further certify that H19 acts not only as an oncogene but also as a tumor suppressor [20,27,33]. Matouk *et al*. found that the knocking-down of H19 RNA resulted in nearly complete attenuation of p57^kip2^ induction in response to hypoxic stress [20]. They further found that H19 was associated with angiopoietin (ANG) and fibroblast growth factor-18 (FGF-18), whose functions are involved in tumor growth and proliferation [20]. To understand the molecular mechanism by which H19 increases gastric cancer cell growth, Yang *et al*. examined whether H19 affects the function of the tumor suppressor p53 [27]. They found that H19 was associated with p53, and that this association resulted in partial p53 inactivation [27].

Contrary to H19, uc001lsz expression level in gastric cancer tissues was found to be markedly lower (Figure 3A). As showed in Figure 3C, the expression of uc001lsz in gastric cancer cell lines (AGS and MGC-803) is lower than that in gastric epithelial cell (GES-1), but there was no significant different between SGC-7901 and GES-1. Maybe the low grade malignancy of SGC-7901 leads to this result. More importantly, a greater association between uc001lsz expression and TNM stage was found (Table 2). These results confirmed uc001lsz as an important player in inhibiting the development of gastric cancer. As we known, many lncRNAs are transcribed close to or within protein-coding loci, which has strengthened the hypothesis that lncRNAs may have *cis*-acting effects within these loci. But uc001lsz may have *trans*-acting effect within its adjacent protein-coding loci. *MUC2*, a member of the mucin protein family of genes, is located next to *UC001LSZ*. The MUC2 is secreted onto mucosal surfaces, where it is secreted from goblet cells in the epithelial lining into the lumen of the stomach [34]. As reported, MUC2 was high expression in gastric cancer [35]. Although uc001lsz seems playing as tumor suppressor gene in gastric cancer and many other types of tumors, it may play different role in prostate cancer where uc001lsz was highly expressed (Figure 3C).

Molecular tumor biomarkers are vital diagnostic and prognostic tools. Our data show that the expression of uc001lsz was aberrant in early gastric cancer and gastric precancerous lesions (Figure 5A). And the extraordinary changes maybe appear in the precancerous lesions (Figure 5B). This investigation indicates that uc001lsz may be a candidate biomarker of gastric cancer.

## Conclusions

In summary, we depict an lncRNA expression profile that associated with gastric cancer. The overexpression of H19 in gastric cancer cell lines and tissues suggests that H19 may be participated in gastric cancer. The reduced expression of uc001lsz in gastric cancer cell lines and tissues, its associations with TNM stage, and its dysregulation in early cancer and precancerous lesions suggest that uc001lsz may have an important role in gastric cancer occurrence and be a potential biomarker for the diagnosis of early gastric cancer.

## Abbreviations

ANG: Angiopoietin; CA19-9: Carbohydrate antigen 19-9; CEA: Carcinoembryonic antigen; Ct: Threshold cycle; DC/CIK: Dendritic cell/Cytokine-induced killer; FGF-18: Fibroblast growth factor-18; GACAT1: Gastric cancer-associated transcript 1; H19: The reciprocally imprinted partner of Igf2; HCC: Hepatocellular carcinomas; HOTAIR: HOX transcript antisense RNA; HOX: Homeobox; HULC: Highly upregulated in liver cancer; IGF2: Insulin-like growth factor 2; IGF2-AS: IGF-IR and IGF-IIR antisense; IGF-IR: Insulin-like growth factor type I receptor; IGF-IIR: Insulin-like growth factor type II receptor; lncRNA: Long non-coding RNA; MALAT-1: Metastasis associated lung adenocarcinoma transcript 1; miRNA: microRNA; MUC2: Mucin 2; NCCN: National comprehensive cancer network; ncRNA: Non-coding RNA; PCR: Polymerase chain reaction; p-TEFb: Positive transcription elongation factor-b; qPCR: Quantitative polymerase chain reaction; RN7SK: RNA, 7SK small nuclear; RT: Reverse transcription; SPSS: Statistical program for social sciences; SUMO1P3: Ubiquitin-like modifier (SUMO) 1 pseudogene 3; TNM: Tumor-node-metastasis.

## Competing interests

The authors declare that they have no competing interests.

## Authors’ contributions

HS conceived of and carried out experiments, analysed and interpreted data and drafted the manuscript; BX and JG conceived of experiments, analysed and interpreted data and wrote the manuscript; WS, GY, XD, ZL, SZ, TX, YX analysed and interpreted data. All authors read and approval the final manuscript.
